# Achieving Optimal Post-Exercise Muscle Protein Remodeling in Physically Active Adults through Whole Food Consumption

**DOI:** 10.3390/nu10020224

**Published:** 2018-02-16

**Authors:** Stephan van Vliet, Joseph W. Beals, Isabel G. Martinez, Sarah K. Skinner, Nicholas A. Burd

**Affiliations:** 1 Center for Human Nutrition, School of Medicine, Washington University, St. Louis, MO 63110, USA; svanvliet@wustl.edu; 2 Division of Nutritional Sciences, University of Illinois at Urbana-Campaign, Illinois, Urbana, IL 61801, USA; jbeals2@illinois.edu; 3 Department of Kinesiology and Community Health, University of Illinois at Urbana-Champaign, Illinois, Urbana, IL 61801, USA; igm2@illinois.edu (I.G.M.); sarahks2@illinois.edu (S.K.S.)

**Keywords:** amino acids, athletic performance, muscle protein synthesis, resistance exercise, endurance exercise

## Abstract

Dietary protein ingestion is critical to maintaining the quality and quantity of skeletal muscle mass throughout adult life. The performance of acute exercise enhances muscle protein remodeling by stimulating protein synthesis rates for several hours after each bout, which can be optimized by consuming protein during the post-exercise recovery period. To date, the majority of the evidence regarding protein intake to optimize post-exercise muscle protein synthesis rates is limited to isolated protein sources. However, it is more common to ingest whole food sources of protein within a normal eating pattern. Emerging evidence demonstrates a promising role for the ingestion of whole foods as an effective nutritional strategy to support muscle protein remodeling and recovery after exercise. This review aims to evaluate the efficacy of the ingestion of nutrient-rich and protein-dense whole foods to support post-exercise muscle protein remodeling and recovery with pertinence towards physically active people.

## 1. Introduction

Exercise is a potent stimulus that, when performed regularly, results in dramatic phenotypic changes to more effectively support exercise performance. For instance, exercise training alters concentrations of proteins responsible for regulating fuel storage [1], energy [2], and force production [3]. These adaptations facilitate greater exercise capacity and performance, but subsequent bouts of exercise are required to preserve the trained phenotype [4,5]. Therefore, a great deal of research has been aimed at the recovery period immediately after a single bout of exercise. Acute bouts of either resistance [6,7] or endurance [8,9] exercise have been shown to increase skeletal muscle proteome remodeling primarily through the synthesis of new proteins in all three major groups (e.g., sarcoplasmic, myofibrillar, collagen/extracellular matrix proteins). Thus, a regular exercise stimulus can result in either a hypertrophic or nonhypertrophic phenotypic response.

While exercise alone is a potent protein remodeling stimulus by increasing muscle protein synthesis rates [6,9], food ingestion throughout post-exercise recovery is necessary to optimize the skeletal muscle adaptive response. It has been clearly established that intravenous infusions or ingestion of free amino acids [10,11,12], or ingestion of isolated protein powders as liquid beverages are potent muscle protein remodeling stimuli [13,14,15]. Moreover, it has been established that the essential amino acids, and leucine in particular [16], are primarily responsible for eliciting a muscle protein remodeling response with protein synthesis (and not breakdown at the muscle level) being the primary metabolic responder in healthy adults [17]. The feeding-mediated potentiation of post-exercise muscle protein synthesis rates is primarily aimed at the myofibrillar protein fraction, and to a lesser extent sarcoplasmic protein synthesis rates [18]. Of course, nonessential amino acid intake is also required to synthesize fully functional muscle proteins, but these amino acids are not generally rate-limiting within a normal eating pattern [19]. In addition, a normal eating pattern generally consists of the regular ingestion of whole foods as opposed to free amino acids or isolated protein powders. As such, it is important to gain an understanding of how whole food ingestion impacts the muscle protein remodeling response to better define protein recommendations to promote post-exercise recovery. 

Our review aims to discuss the effectiveness of nutrient-rich and protein-dense food ingestion to achieve protein requirements and support post-exercise muscle protein remodeling and repair for physically active individuals. Indeed, lifestyle today has become increasingly sedentary to support an obese phenotype. To this end, it is especially important to consider how exercise impacts protein nutritional requirements as both diet and exercise manipulations are important to maximize a healthy phenotype. Given the current research gap for protein nutrition for contact sport, middle-distance, or endurance exercise, much of our discussion is based on resistance exercise studies. This is a recognized shortcoming as sport- and exercise-mode specific protein recommendations may be required to maximize the immediate post-exercise muscle protein remodeling response [20,21].

## 2. Protein Recommendations for Physically Active Adults

Protein ingestion during both the immediate (i.e., first 1–5 h) and prolonged post-exercise recovery window (i.e., 5–72 h) is important for all physically active adults to optimize the skeletal muscle adaptive response. Aside from timing [22] and amount [23,24], the ingested protein source is a fundamental consideration when the goal is to optimize post-exercise muscle protein remodeling and repair, especially with the increasing popularity of restrictive dietary patterns (e.g., vegetarian, ketogenic, paleolithic etc.) that might narrow food protein selection. As such, identifying the protein source that is most suitable to optimize muscle anabolic action is a question of both practicality and feasibility and may depend on nutritional (amino acid profile) and non-nutritional factors such as cost, availability, and taste preferences. 

Recent position stands from the Academy of Nutrition and Dietetics, Dietitians of Canada and the American Colleges of Sports Medicine [25], and the International Society of Sports Nutrition [26] recommend a protein intake of 1.2–2.0 g·kg body weight (BW)^−1^·day^−1^ to support metabolic adaptations and repair and remodeling of skeletal muscle tissues in healthy physically active adults. Morton et al. [27] have demonstrated that a dietary protein intake of ~1.6 g·kg body weight (BW)^−1^·day^−1^ is maximal to support resistance-exercise-induced strength and lean mass gain in healthy adults in energy balance. What is noteworthy is that resistance exercise improves muscle protein net balance for at least 2 days in the postabsorptive state [7]. Moreover, regular resistance exercise training results in increased whole body nitrogen retention when compared with the untrained state [28]. Finally, acute resistance exercise enhances the dietary amino acid sensitivity of myofibrillar protein synthesis rates for at least 1 day into recovery [18]. With these factors in mind, it is evident that resistance exercise is fundamentally anabolic and may actually shift the utilization of dietary amino acids towards muscle protein synthesis and thus a greater ratio of circulating amino acids are being retained by the body’s largest protein pool (skeletal muscle) in both the fasting and fed states. Hence, a person engaged in a regular resistance exercise training program is likely at the lower end of required protein intakes to optimize muscle adaptive response when compared with those engaged in other exercise activities. For example, Kato et al. [29] have demonstrated that protein requirements are slightly elevated for endurance-trained adults consuming an energy-balanced diet (~1.8 g·kg body weight (BW)^−1^·day^−1^). 

It is evident that other factors, besides exercise type, intensity, and duration, may also influence daily protein requirement and muscle mass regulation. For example, it has been suggested that protein intakes up to 1.6–3.1 g protein·kg BW^−1^·day^−1^ may be required for physically active adults during periods of decreased caloric intake for lean mass maintenance [26]; however, these recommendations will fluctuate based on the severity of the energy restriction protocol and the type of exercise training performed. It has also been put forward that higher dietary protein intakes (3.0 g protein·kg BW^−1^·day^−1^) may strengthen the immunity of individuals engaging in intense training bouts [30]. These data highlight that muscle vs. other body tissues may have differential protein ‘requirements’ to optimize the adaptive response. Moreover, it has been established that it is more beneficial to evenly distribute the total daily protein intake throughout the day such that ~0.25–0.40 g protein·kg BW^−1^ per meal (or 20–40 g depending on body mass or age) [14,24,31] is ingested to optimally stimulate post-exercise muscle protein synthesis rates. This is relevant as the pattern of regular dietary protein intake may influence daily protein requirements with a spread distribution pattern being superior to a skewed protein eating pattern (i.e., the majority of protein calories ingested at the dinner meal time). Regardless, there is an overwhelming consensus that clearly demonstrates that the protein Recommended Dietary Allowance (RDA; set at 0.8 g protein/kg/day) is not set to optimize post-exercise muscle protein remodeling and recovery—a point that has been reviewed in detail elsewhere [32]. 

It is not surprising that the optimal amount of protein to consume to support a robust post-exercise muscle protein remodeling response is elevated against the protein RDA [33]. Specifically, the RDA was set to prevent protein deficiencies in a sedentary population within a safety margin. However, the above data illustrate that higher protein intakes are likely required to support an optimal skeletal muscle adaptive response and are mildly concerning from a protein sustainability and diet quality standpoint. For instance, the amount of daily dietary protein required to maximize muscle protein remodeling throughout a prolonged recovery period (e.g., ~2–3 day [7,34]) would be placed at ~2.0 g protein·kg BW^−1^·day^−1^ based on five meal times. These elevated protein recommendations have the potential to displace other vital nutrients and negatively impact diet quality. Specifically, with increased food processing technologies, the use of derivative protein products (i.e., whey, casein, soy, and pea protein supplements) to meet daily protein requirements have gained widespread popularity among physically active individuals due to convenience [35] and touted ergogenic properties [36]. Notwithstanding, the consumption of isolated protein supplements, often in combination with carbohydrate supplements to facilitate muscle glycogen resynthesis rates, are likely important for certain sports. For instance, it is quite common for some athletes to participate in multiple training sessions in a single day with limited recovery time in between sessions [37]. This is common in competitions for many sports (i.e., swimming, windsurfing, water polo, football, track and field) wherein competitors may participate in several heats or events in a single day. In addition, many physically active individuals (in particular amateur and recreational athletes) must balance work life with exercise, which may limit the available time for meal preparation. Finally, it may be difficult for some people to obtain their daily protein intake (upwards to ~2.0 g protein·kg BW^−1^·day^−1^ based on some data) exclusively from whole foods due to the ‘volume’ of food that is required to meet such protein intakes. Given these concerns, protein supplements may have a complementary role in the diet of physically active individuals as they require minimal preparation and handling, may be more rapidly digested and absorbed [13,38], and provide a high amount of protein relative to their total volume (e.g., a 300 mL drink containing 25 g of dietary protein) when compared with whole food protein sources [39,40,41]. However, an important consideration when opting for protein supplements is to ensure the supplement is of high quality (i.e., the nutrient composition is in accordance with the label) and safe (i.e., free of banned substances and toxins). Although the risk for presence of banned substances in protein supplements is low when compared to other dietary supplements [42], there have been accounts of protein supplements containing heavy metals (e.g., cadmium and arsenic above safe levels) or being ‘spiked’ with low-cost free amino acids (such as glycine and glutamine) to bring up the nitrogen content of the product to ‘artificially’ meet the stated protein content [43]. As such, it is important to obtain protein supplements from a reputable source that is NSF Certified for Sport^®^ and/or has other third-party testing certification on the label. Alternatively, when safety/quality of protein supplements is a concern, obtaining dietary protein from whole foods as much as possible is clearly preferred. 

## 3. Isolated Protein Sources and Post-Exercise Muscle Protein Remodeling

Biolo et al. [10], in a seminal study, demonstrated a synergy between resistance exercise and hyperaminoacidemia on the stimulation of muscle protein synthesis rates during post-exercise recovery in healthy adults. These findings have since been recapitulated using the ingestion of free AA or isolated protein sources in a variety of resistance exercise settings [18,39,44,45]. The interactive effect between resistance exercise and dietary-protein-derived amino acid availability on the stimulation of muscle protein synthesis rates appeared to be largely due to the essential AA content of the ingested protein source with the leucine content being exceptionally relevant for an optimal response [46]. The latter notion lead to a hypothesis often referred to as the leucine threshold or trigger hypothesis [13]. This hypothesis suggests that the peak amplitude of blood leucine concentrations after protein ingestion is a primary determinant of a robust postprandial muscle protein synthetic response. However, this hypothesis was developed based on the ingestion of isolated protein sources, and not nutrient-dense whole protein foods. For example, the ingestion of isolated protein sources generally results in a rapid peak leucinemia (within 30 min) in healthy adults [13,38,47]. However, protein-dense whole food ingestion results in slower protein digestion and amino acid absorption rates with a peak dietary amino acid rise at ~60–120 min of the postprandial period, depending on whether the whole food source is ingested in liquid form (peak around ~60 min) or in a solid form (peak around ~120 min) [39,41,48,49]. Thus, the leucine trigger/threshold hypothesis to support an optimal post-exercise muscle protein synthetic response may only be relevant for the ingestion of isolated protein sources and not whole food sources of dietary protein.

## 4. Whole Food Ingestion and Post-Exercise Muscle Protein Remodeling

As much of the available work regarding post-exercise recovery nutrition has been conducted using isolated protein sources with a special emphasis on whey protein [13,15,38,47,50,51,52,53,54,55], there is a clear research gap related to how nutrient-rich, protein-dense foods are impacting ingested protein dose–response curves and overall protein requirements for post-exercise muscle protein remodeling and repair. Moreover, current protein meal recommendations to maximize post-exercise muscle protein synthesis rates are generally resistance-exercise-centric. Resistance exercise is generally associated with short periods of muscle contractile activities with less of an increase in exercise-induced whole-body leucine oxidation rates when compared with endurance exercise activities. Recent work has shown that typical protein meal recommendations for resistance-trained adults (~0.25 g protein/kg per meal) was not sufficient to support a positive whole body net leucine balance, after accounting for exercise-induced whole body leucine oxidative loss, during recovery from 1 h of treadmill running in endurance-trained young men [21]. Thus, it is evident that more work is required to more optimally define protein meal recommendations in a more sport-specific manner. At the moment, there is very little information regarding how endurance exercise intensity and duration impacts the ingested protein dose–response curves on the post-exercise muscle protein synthetic response [21,56].

Table 1 lists the serving size of commonly ingested protein-rich whole foods to achieve 30 g of protein in a meal to, in theory, optimally support post-exercise muscle protein remodeling over a prolonged recovery period. This ingested protein amount (30 g) should increase postprandial muscle protein synthesis rates with minimal resultant increase in whole body amino acid oxidation rates. It is important to note that this 30 g protein value is largely based on the data obtained from dose–response curves on muscle protein synthesis rates generated based on the ingestion of isolated protein sources after resistance exercise and does not take into account the potential of prolonged practices or endurance exercise intensity and duration to directly impact these meal protein (amino acid) requirements. Moreover, Table 1 does not take into account the potential of different foods and their associated whole food matrix effects to interact and create a food synergy to differentially modulate post-exercise muscle protein synthesis rates. This notion is discussed in more detail below.

For coaches and clinicians, typical meal patterns must be considered when making recommendations for post-exercise recovery nutrition. For example, proteins in the diet of Western athletes are predominantly of whole food animal origin and include sources of meat, dairy, eggs, and seafood with lower amounts of protein obtained from grains and legumes [58,59]. On the other hand, many African and Asian athletes obtain a large portion of their daily protein from plant-based whole foods such as grains and legumes [60,61,62]. In general, protein from animal sources (i.e., dairy, beef, chicken, pork, etc.) is considered higher quality than plant protein [63], as animal proteins contain proportional amounts of all the essential amino acids for optimal support of skeletal muscle protein remodeling [64]. However, when multiple plant sources are combined (e.g., wheat or rice, combined with bean or pea), thereby completing the amino acid profile [64], and are consumed in sufficient quantities (>1.2 g protein·kg BW^−1^·day^−1^) [65], these plant-based proteins may be effective for supporting post-exercise muscle protein remodeling and repair. Nevertheless, much work is needed to confirm the capacity of plant-based whole food sources (containing multiple macro- and micronutrients, fiber, etc.) to support skeletal muscle protein remodeling as the majority of research on whole food ingestion to support post-exercise skeletal muscle protein remodeling and recovery has focused on animal-based sources such as beef, eggs, and dairy.

Besides food preferences, the age of the individual is a further point of consideration for optimal daily protein intakes for physically active adults. For example, it has been shown that aging muscles may require higher dietary protein intakes to recover from intense workouts [66]. It has also been demonstrated that younger individuals can maximize muscle protein synthesis rates at lower ingested protein intakes (~0.25 g protein·kg BW^−1^ per meal) than older individuals (~0.40 g protein·kg BW^−1^ per meal) [24]. These protein meal recommendations for younger and older adults were based on the ingestion of isolated protein sources. It is currently not clear how protein meal recommendations based on isolated protein sources relate to whole food protein sources. Given the high prevalence of supplement use in active older adults [67,68,69], it may be beneficial to obtain at least part of daily protein requirements through protein supplements for older adults. However, when dietary supplement avoidance is preferred, it may be recommended that a greater amount of dietary protein is obtained from liquid whole food sources, such as milk and yoghurt. Liquid sources of protein are potentially less satiating than solid whole food sources [70,71] and in turn may allow the individual to consume more of these foods [72]. 

The ingestion of protein-dense whole foods to stimulate post-exercise muscle protein remodeling and repair may provide benefits apart from directly stimulating synthesis of muscle proteins. For instance, many whole food protein sources are quite nutrient dense, which provides unique benefits for athletes in certain sports. For instance, weight-class athletes (i.e., gymnasts, boxers, weightlifters, bodybuilders, etc.) often have periods of reduced caloric intakes, which warrants maximizing nutrient quality of each meal or snack. In addition, the satiating effects of solid whole food protein meals are greater than those of liquid protein meals [70,71]. Hence, the ingestion of the majority of dietary protein from solid whole foods during periods of reduced caloric intake may be beneficial to achieve the desired weight and body composition goals. Furthermore, limited nutritional knowledge and common misconceptions [73,74,75] may place physically active individuals at risk for health problems and/or performance decrements. As such, recommendations to consume protein-rich whole foods will help increase the nutrient density of the diet and may positively impact health, body composition, and athletic performance. Besides providing high-quality protein, whole foods also provide significant amounts of beneficial, and often essential, nutrients as part of their natural food matrix (i.e., vitamins, minerals, omega-3 fatty acids, growth factors, peptides, etc.) [76,77,78] that likely play a role in exercise recovery and overall health. In addition, there is a possibility that there is an interactive effect of the food matrix (i.e., food synergy) on protein metabolic responses such that nonprotein food components may also have a role in supporting the post-exercise muscle protein synthetic response. 

For instance, several studies have demonstrated that bovine milk provides benefits beyond its constituent proteins (e.g., ~20% whey and 80% casein by total protein mass). Milk consumption after exercise has been demonstrated to attenuate exercise-induced muscle damage [79,80], and limited decrements in exercise performance (e.g., slower sprint efforts) with repeated exercise [79]. Similar findings have been made with chocolate milk, which has been shown to reduce muscle soreness and sustain exercise performance with repeated bouts of exercise [81,82]. The beneficial effects of consuming (chocolate) milk on exercise performance may be the result of co-ingestion of protein and carbohydrate [83] which enhances glycogen repletion [84] while protein supports muscle protein remodeling (i.e., protein synthesis) [39]. Milk, aside from providing 8 grams of protein and 12 g of carbohydrate per cup (240 mL), also appears to be an excellent choice for post-exercise rehydration as it has a comparable electrolyte content to commonly used sports drinks [85]. In particular, evidence exists that milk-based drinks are as effective, or even more effective, than commercially available sports drinks for rehydrating after exercise [86,87,88]. Dairy products are also excellent sources of micronutrients important for bone health (i.e., calcium, vitamin D, and phosphorus) [89]. This may especially be important for females who are at higher risk for vitamin and mineral deficiencies (i.e., iron, vitamin D, and calcium) then men [90]. Thus, a food matrix that is rich in dietary protein, calcium, fatty acids, sugars, etc., likely has multiple benefits towards post-exercise recovery.

Various research groups have also demonstrated the effectiveness of whole food protein sources to stimulate post-exercise muscle protein remodeling and repair. For example, it has been demonstrated that beef can effectively support the post-exercise rise in muscle protein synthesis rates [39,48,91]. Moreover, beef ingestion may be more effective in stimulating post-exercise muscle protein synthesis rates than an isonitrogenous soybean-based meat replacement [92]. Recently, it has been shown that the ingestion of beef and skim milk (both providing 30 g of protein) were equally as effective in stimulating post-exercise muscle protein synthesis rates throughout a 0–5 h post-exercise period [39]. Similarly, egg ingestion has been shown to be effective at stimulating the post-exercise muscle protein synthetic response [41]. Additionally, there is emerging evidence that the food matrix in which the protein is consumed may have a direct influence on the post-exercise muscle protein synthetic response in healthy young adults.

For example, Elliot et al. [93] previously showed greater skeletal muscle amino acid uptake across the leg, and presumably greater net leg muscle protein synthesis, after consumption of whole (3.25% fat) vs. skim milk (0.5% fat) during recovery from resistance exercise. Similarly, we recently observed a greater post-exercise muscle protein synthetic response with consumption of whole eggs when compared with the consumption of egg whites, despite being matched for protein content in healthy young resistance-trained men [41]. What is noteworthy is that egg white ingestion showed a more rapid rise in leucinemia/aminoacidemia [41], which has been historically considered a key determinant of the subsequent postprandial rise in muscle protein synthesis rates [11,13,94]. Moreover, the energy content of the ingested whole eggs likely did not contribute to the superior post-exercise muscle protein synthetic response vs. egg white ingestion [41]. The above examples of whole foods (milk and eggs) contain a host of potentially anabolic compounds within their food matrices that may exert functional and biological activity in the human body [95,96]. For instance, several vitamins (e.g., A, D, E), minerals (e.g., zinc and selenium), and dietary fats (e.g., palmitate and Omega 3 highly unsaturated fatty acids (n-3 HUFAs)) are primarily contained in the yolk and milk fat (Table 2). Indeed, several of these vitamins and minerals have been demonstrated to mediate translational control of muscle protein synthesis by activating the mTORC1-signaling pathway either in vitro or in vivo in animals [97,98,99,100,101]. Furthermore, saturated fatty acid palmitate, highly present in animal fats, can acutely activate mTORC1 in vitro [102]. 

Moreover, there is evidence that the n-3 HUFAs—eicosapentaenoic acid (EPA) and docosahexaenoic acid (DHA)—are capable of modulating the postprandial muscle protein synthetic response. These dietary fats are most commonly found in fatty seafood [57], but also can be found in meats [103], dairy (EPA only) [104], and eggs [105], particularly when pasture-raised. These HUFAs offer a potential benefit for enhancing postprandial muscle protein synthesis rates [106,107], at least when protein intake is suboptimal. For example, preliminary evidence in strength-trained young men suggests that n-3 HUFA supplementation does not alter muscle protein synthesis rates in response to a single meal if adequate protein (e.g., 30 g) is consumed [108]. In contrast, there is some evidence to suggest that long-term supplementation of n-3 HUFAs in combination with resistance exercise may augment mass in older adults [109,110] and attenuates exercise-induced muscle soreness [111] and oxidative stress [112] in young adults. This information may be relevant for those who engage in repeated bouts of exercise with short recovery times in between training bouts or whose dietary intake of n-3 HUFAs is inadequate due to low caloric intake. However, clearly more research is required to identify if a protein-rich food matrix is providing a food synergy to enhance the post-exercise muscle protein synthetic response. Collectively, the ingestion of protein-rich whole foods is capable of supporting post-exercise muscle protein remodeling by stimulating muscle protein synthesis rates, and may provide a lifestyle strategy of improving the overall diet quality of exercising adults. 

## 5. Conclusions

Identifying the most effective source of protein to increase post-exercise muscle protein synthesis rates for active individuals will depend on both nutritional needs and non-nutritional factors such as availability, cost, and preference. Thus, protein nutrition for performance is personalized. However, the ingestion of whole foods, which contain a food matrix rich in dietary protein, vitamins, minerals, and other macronutrients, to stimulate post-exercise muscle protein remodeling may also provide additional benefits, such as improvements in overall diet quality. In addition, there is evidence that non-protein food components, and the matrix of different foods, may also have a direct influence on changes of post-exercise muscle protein synthesis rates. However, obtaining dietary protein exclusively from whole foods may not always be convenient, or feasible, due to a variety of reasons including cost, palatability, competitive or training schedule, and volume of whole food that needs to be consumed to meet protein requirements. In such cases, protein supplements remain a convenient complementary nutritional strategy for physically active adults to meet protein recommendations at select meals throughout the day, while still providing dietary amino acids to stimulate skeletal muscle protein remodeling and repair.

## Figures and Tables

**Table 1:** 30 g Servings of Protein from Whole Food Protein Sources ^1^.

Protein Source	30 g PRO
**Meats**	
Chicken ^2^, Beef ^2^, Pork ^2^, Turkey ^2^	126 g (4.5 oz.)
**Eggs**	
Whole eggs ^2^	250 g (5 large eggs)
Egg whites ^2^	264 g (8 large eggs)
**Seafood**	
Finfish ^2^, Shellfish ^2^, Crustaceans ^2^	126 g (4.5 oz.)
**Dairy**	
Milk, Yogurt	750 mL (3 cups)
Greek Yogurt	298 g (1.8 cups)
Cheese	140 g (5 oz.)
Cottage Cheese	290 g (2 cups)
**Nuts, Seeds, Legumes**	
Beans ^2^ and Peas ^2^	410 g (2.5 cups)
Nuts ^2^ and Seeds	725 g (5 cups)
Quinoa ^2^	682 g (3.7 cups)
**Grains**	
Corn ^2^	880 g (5 cups)
Wheat bread ^2^	241 g (7.5 slices)
Rice	1095 g (5.4 cups)
**Soy Products**	
Soybeans ^2^	172 g (1 cup)
Soymilk	911.3 mL (3.8 cups)
Tempeh ^2^	149 g (0.9 cup)
Tofu ^2^	372 g (1.5 cups)

^1^ Based on data from [57]. ^2^ Amounts are expressed in cooked weights.

**Table 2:** Nutrient comparison of ‘whole’ vs. ‘processed’ food sources matched for 30 g protein ^1^.

Nutrient (% Daily Value )	Protein Source					
	Whole Milk	Skim Milk	Whole Egg	Egg White	Tempeh	Soy Isolate
Protein, g	30	30	30	30	30	30
Carbohydrates, g	45	45	2	2	12	0
Fat, g	31	1	22	0	17	1
Palmitic acid, g	7.9	0.2	5.3	0	3.3	0.1
Docosahexaenoic acid, mg	0	0	139	0	0	0
Calcium, mg	1076 (108%)	1086 (108%)	134 (13%)	2 (2%)	145 (15%)	61 (6%)
Iron, mg	0.27 (2%)	0.29 (2%)	4.18 (23%)	0.22 (1%)	3.21 (18%)	4.93 (27%)
Magnesium, mg	98 (25%)	95 (24%)	29 (7%)	30 (7%)	116 (29%)	13 (4%)
Phosphorus, mg	800 (114%)	899 (128%)	473 (67%)	41 (6%)	381 (54%)	264 (38%)
Selenium, mg	35 (50%)	27 (40%)	73 (104%)	55 (78%)	0 (0%)	0 (0%)
Sodium, mg	410 (18%)	374 (16%)	339 (15%)	457 (20%)	21 (1%)	342 (15%)
Potassium, mg	1389 (40%)	1257 (36%)	330 (9%)	449 (13%)	604 (17%)	28 (1%)
Zinc, mg	3.74 (27%)	3.52 (25%)	3.08 (22%)	0.08 (1%)	2.37 (17%)	1.37 (10%)
Vitamin A, IU	1543 (30%)	0 ^2^ (0%)	1291 (26%)	0 (0%)	0 (0%)	0 (0%)
Vitamin B6, mg	0.33 (13%)	0.34(13%)	0.40 (16%)	0.01 (1%)	0.30 (12%)	0.03 (1%)
Vitamin B12, μg	4.45 (74%)	4.29 (72%)	2.13 (36%)	0.25 (4%)	0.21 (4%)	0 (0%)
Vitamin D, IU	19 (2%)	0 (0%)	196 (25%)	0 (0%)	0 (0%)	0 (0%)
Vitamin E, mg	0.67 (3%)	0.09 (1%)	2.51 (13%)	0 (0%)	0 (0%)	0 (0%)
Vitamin K, μg	2.9 (4%)	0 (0%)	0.7 (1%)	0 (0%)	0 (0%)	0 (0%)
Pantothenic Acid	3.5 (71%)	3.2 (63%)	3.6 (73%)	0.5 (10%)	0.6 (14%)	0 (0%)

^1^ Data from USDA National Nutrient Database for Standard Reference, Release 28 (Slightly revised) [57]. ^2^ 1816 IU if fortified with retinyl palmitate.
